# Challenges of Acute Ischemic Stroke Treatment in Orally Anticoagulated Patients via Telemedicine

**DOI:** 10.3390/jcm10091956

**Published:** 2021-05-02

**Authors:** Jordi Kühne Escolà, Simon Nagel, Verena Panitz, Tilman Reiff, Alexander Gutschalk, Christoph Gumbinger, Jan Christoph Purrucker

**Affiliations:** Department of Neurology, Heidelberg University Hospital, 69120 Heidelberg, Germany; jordi.kuehneescola@gmail.com (J.K.E.); simon.nagel@med.uni-heidelberg.de (S.N.); verena.panitz@med.uni-heidelberg.de (V.P.); tilman.reiff@med.uni-heidelberg.de (T.R.); alexander.gutschalk@med.uni-heidelberg.de (A.G.); christoph.gumbinger@med.uni-heidelberg.de (C.G.)

**Keywords:** cerebral stroke, cerebrovascular disease, oral anticoagulation, telemedicine, teleneurology

## Abstract

Background: Managing acute ischemic stroke (AIS) in patients receiving treatment with vitamin K antagonists (VKA) or non-VKA oral anticoagulants (NOACs) is difficult and the challenge this poses for stroke telemedicine remains unexplored. Methods: We analyzed data from a random sample (*n* = 1500) of all teleneurological consultations conducted between July 2015 and December 2017. Management of patients suffering AIS with and without prior oral anticoagulation treatment was characterized, including potential vs. actual treatment with intravenous thrombolysis (IVT) and reasons for withholding it. Results: *n* = 359 patients had suffered an AIS, of whom 63 (17.5%) were under treatment with oral anticoagulants (VKA, *n* = 24; NOAC, *n* = 39). Administration of IVT was more common in patients who had not received prior oral anticoagulation treatment (20.3% vs. 3.2%, *p* < 0.001). NOAC intake was the primary reason for withholding IVT in 37% of orally anticoagulated patients who were found potentially eligible for IVT. Furthermore, patients under oral anticoagulation tended to be transported to the comprehensive stroke center more often (23.8% vs. 13.9%, *p* = 0.056). Conclusions: AIS in patients on oral anticoagulation treatment is a frequent reason for telestroke consultation, and NOAC intake constitutes an important barrier to administering IVT.

## 1. Introduction

Non-vitamin K antagonist oral anticoagulants (NOACs) have become the therapy of choice for primary and secondary prevention of ischemic stroke and systemic embolism in patients with nonvalvular atrial fibrillation. Nevertheless, while the relative risk of hemorrhagic strokes has been halved by using NOACs as compared to warfarin, a vitamin K antagonist (VKA), the rate of ischemic stroke has remained largely unchanged [1]. Consequently, acute ischemic stroke (AIS) develops frequently despite oral anticoagulation [2,3]. Current evidence about the optimal treatment strategies in patients with AIS under therapy with NOACs is still limited [4]. Determining the coagulation status by relying on the time since an NOAC was last taken is limited by interindividual variability [5], including drug-drug interactions and individual genetic predispositions. Substantial prospective data are missing regarding the safety of measuring NOAC concentrations before thrombolysis, and uncertainty exists about the optimal threshold below which intravenous thrombolysis can be performed safely [4,6]. However, according to current recommendations, such an approach can be considered in patients with a relevant clinical deficit and in whom endovascular thrombectomy is not indicated or is not available [7,8]. While management of patients with AIS under oral anticoagulant therapy is challenging even in comprehensive stroke centers (CSC) [9,10], no data exist on the relevance of the condition for telestroke medicine.

We therefore aimed to describe how AIS is currently managed in patients under therapy with oral anticoagulation in the setting of teleneurology and to identify barriers to acute reperfusion treatment in these patients.

## 2. Methods

### 2.1. Study Design and Setting

The Heidelberg Teleneurology Network consists of seven primary stroke centers (PSC) and one CSC, covering a large geographic area of parts of three federal states in southwestern Germany. The primary stroke centers treat between 150 and 780 strokes per year (AIS, transient ischemic attack, and intracerebral hemorrhage). As of 2021, six of seven PSC have officially been certified by the German Stroke Society or by a stroke unit working group of a federal state, which means they must adhere to large number of standards and meet quality assurance parameters for stroke care. Local patient management is handled by emergency physicians, usually from internal medicine departments, and teleneurological expertise is provided 24/7 by an experienced stroke neurologist via real-time videoconferencing and teleradiology. In addition, five of seven sites have on-site neurologists, including at small neurology departments in two clinics that handle patients with acute conditions during regular working hours. Teleneurological consultations are requested by the spoke-centers in every patient with suspected stroke, irrespective of the time-window, medical history, previous medication, symptom severity, or health-insurance coverage. After the initial consultation, recommendations regarding further diagnostic testing and the acute therapeutic approach are made by the teleneurological consultant, including decisions to transfer to the CSC. A study on diagnostic accuracy of our teleneurological stroke consultations was recently published in [11]. In patients with AIS, options for recanalization therapy include intravenous thrombolysis (IVT; usually administered at the local PSC), endovascular treatment (EVT; available after transfer to the CSC only), or a combination of the two as drip-and-ship.

To avoid supervisor bias, we aimed to analyze a random sample of 25% of all teleneurological consultations between July 2015 and December 2017. Given a total of *n* = 6220 consultations, the resulting number was rounded down to the next hundred and, thus, *n* = 1500 consultations were screened.

Ethical approval was obtained from the ethics committee of the Medical Faculty of the University of Heidelberg (S-007/2018).

### 2.2. Data Acquisition

Digital hospital archives from all participating sites were screened for teleneurological consultation records and final discharge reports. Demographic characteristics, medical history, imaging data, and information on acute and post-acute stroke treatment were extracted. Premorbid functional status, categorized as premorbid modified Rankin scale score (pmRS, ranging from 0 [no disability] to 5 [severe disability]), and National Institutes of Health stroke Scale (NIHSS) score at admission and discharge, were obtained. Reasons for withholding potential treatment with IVT were recorded for all patients who were admitted to the PSC within 4.5 h after stroke symptom onset. Data were processed using Microsoft Excel (Microsoft Corp., Redmond, WA, USA).

### 2.3. Statistical Analysis

Descriptive statistics were used to characterize baseline data and management of AIS patients. Continuous data are presented as median (interquartile range) and absolute and relative frequencies (counts and percentages) are shown for categorical data. The Kolmogorov–Smirnov test was used to ascertain distribution of data. Patients who were under treatment with oral anticoagulants were compared to those who were not receiving oral anticoagulation treatment at the time of presentation. Fisher’s exact test or nonparametric Mann–Whitney *U* test were used according to the level of measurement. We calculated a binary logistic regression analysis with transfer as the dependent variable and explanatory variables of the univariate between-group comparison (OAC+ vs. OAC-) with a *p* value < 0.05 (method: enter). We excluded medical history of atrial fibrillation, deep-venous thrombosis or pulmonary embolism due to the strong correlation with OAC-use. All statistical tests were two sided, and *p* values of < 0.05 were considered statistically significant. No adjustment was made for multiple testing. Statistical analysis was performed using SPSS, versions 26 and 27 (IBM Corp., Armonk, NY, USA).

## 3. Results

**Figure 1:**
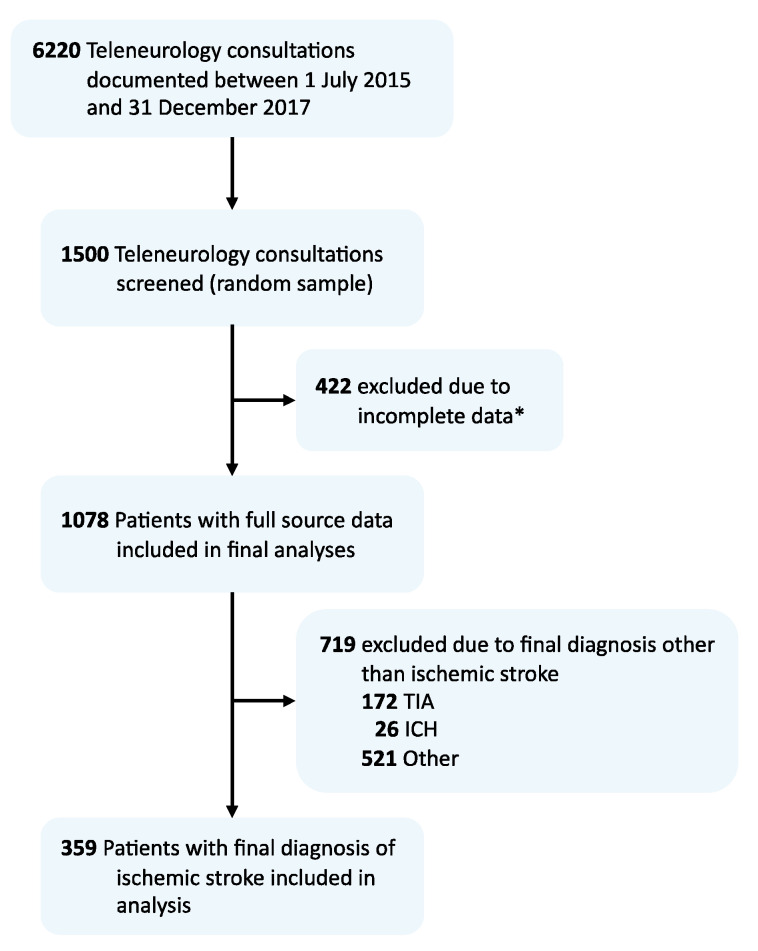
Flow chart depicting the study population. Abbreviations: TIA, transient ischemic attack; ICH, intracerebral hemorrhage. * Of 422 consultations excluded from analysis, *n* = 164 patients were excluded due to incomplete discharge data transfer to hub. In *n* = 228 patients, discharge data sets were incomplete or missing due to patients being transferred to third-party CSC, discharge against medical advice, errors linking archived documents to teleneurological consultations, or changes in hospital information system documentation standards (1 center). Another *n* = 30 consultations were ultimately canceled or performed with no written standardized documentations.

Full source data were available for *n* = 1078 of 1500 consultations (71.9%), and *n* = 359/1078 (33.3%) had a final diagnosis of AIS (see Figure 1). Here, *n* = 63/359 (17.5%) suffered an AIS while being anticoagulated (VKA, *n* = 24; NOAC, *n* = 39). Compared to patients who did not receive prior oral anticoagulation treatment, orally anticoagulated patients were older (median age 79.8 years, IQR 75.3–85.3 vs. 75.4, 64.1–81.6; *p* < 0.001) and presented with more comorbidities (see Table 1). In patients receiving prior anticoagulation, prevalence of atrial fibrillation (84.1% vs. 11.5%; *p* < 0.001), prior deep vein thrombosis or pulmonary embolism (20.6% vs. 2.7%; *p* =< 0.001), and prediagnosed malignancy (23.8% vs. 8.1%; *p* < 0.001) were observed more frequently and the premorbid grade of disability (median premorbid mRS 2, IQR 1–3 vs. 1, 0–3; *p* = 0.002) was higher. Median NIHSS at admission was also higher in orally anticoagulated patients (median NIHSS 6, IQR 3–13 vs. 4, 2–9; *p* = 0.014). In-hospital mortality was 5% (all non-anticoagulated).

### 3.1. Coagulation Status

A total of 39 patients had received prior NOAC treatment (apixaban, *n* = 17; rivaroxaban, *n* = 11; dabigatran, *n* = 10; edoxaban, *n* = 1). Specific quantitative coagulation testing was only available in *n* = 8 cases (21%) after the patients were transferred to a comprehensive stroke center. In two of these patients, NOAC concentrations ≤ 30 ng/mL were found. Of 24 patients under prior VKA treatment, more than half (*n* = 13) had a subtherapeutic INR < 2.0 at admission and one-third (*n* = 8) had an INR < 1.7. Point-of-care testing was not performed. In *n* = 5/63 patients (8%) under therapy with oral anticoagulants, medication history was uncertain at the time of presentation but NOAC intake within 48 h before presentation was revealed in the further course of inpatient care.

### 3.2. Acute Ischemic Stroke Management

A total of *n* = 81/359 patients (22.6%) received IVT and/or endovascular therapy (see Table 2). The rate of patients who were admitted within 4.5 h after symptom onset did not differ between orally anticoagulated patients and those without prior anticoagulation treatment (50.8% vs. 44.6%, *p* = 0.405). Characteristics of patients admitted within the 4.5 h time-window are summarized in Table A1 and Table A2 (Appendix A). Briefly, patients in the oral anticoagulation group who presented in the ≤ 4.5 h time-window had more previous arterial hypertension, more previous stroke/TIA, and an increased length-of-stay compared to no-anticoagulation, but functional status was not different compared to patients without prior anticoagulation.

IVT was more common in patients who had not received treatment with oral anticoagulants (3.2% vs. 20.3%, *p* < 0.001). Large vessel occlusion was found in a total of *n* = 57 patients (15.9%), and *n* = 30 (8.4%) received endovascular therapy, with no difference between patients with and without prior oral anticoagulation. Patients under oral anticoagulation tended to be transported to the CSC more often (23.8% vs. 13.9%, *p* = 0.056). Binary logistic regression analysis confirmed a trend towards higher odds of secondary transfer due to OAC use (OR 2.306, 95% 0.958–5.552, *p* = 0.062) (Table A3). In addition, a vascular imaging examination had been performed at the local PSC in *n* = 33/56 patients who were then transferred to the CSC in an acute situation (40.0% vs. 65.9%, *p* = 0.125). Further treatment characteristics such as admitting ward, length of stay, and discharge facility were evenly distributed between the two groups, as were complications, including intracerebral hemorrhage (3% overall). Two patients suffered intracerebral hemorrhage in the prior OAC group, none of which was associated with IVT or EVT. While a cardioembolic etiology was more frequently identified in orally anticoagulated patients (66.7% vs. 23.3%, *p* < 0.001), large artery atherosclerosis was more common in patients without prior anticoagulation (9.5% vs. 26.4%, *p* = 0.003). Stroke etiology remained undetermined in one-third of cases (*n* = 118), which was more common in patients without prior anticoagulation (11.1% vs. 37.5%, *p* < 0.001).

### 3.3. Reasons for Withholding Intravenous Thrombolysis

**Figure 2:**
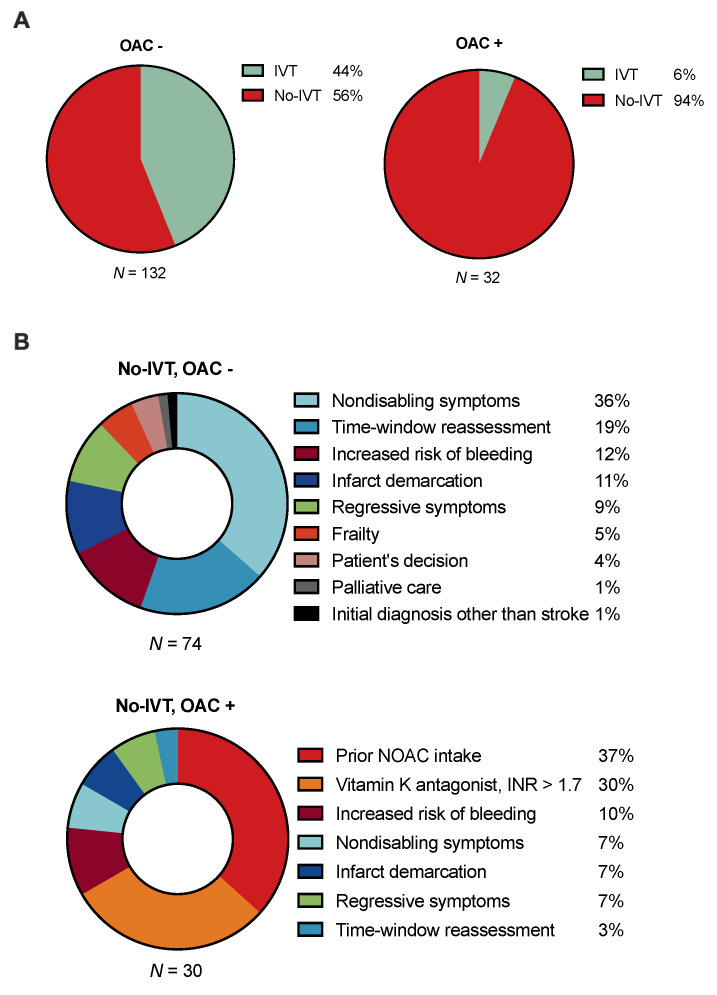
(**A**) Relative proportion of patients with ischemic stroke presenting within the 4.5-h time window receiving intravenous thrombolysis (IVT) with and without prior oral anticoagulation (OAC) who were considered eligible in first teleneurological assessment. (**B**) Reasons for not performing IVT in patients with and without OAC. NOAC, non-vitamin K antagonist oral anticoagulant. Increased risk of bleeding includes prior ICH (*n* = 3), esophageal varices, active lung cancer with active bleeding, gastrointestinal ulcer, recent spinal infiltration, hemorrhagic transformation, recent GI bleeding, and acute fracture (all *n* = 1). Percentages do not round up to exactly 100% due to rounding.

Of all 359 patients, *n* = 164 (45.7%) were admitted to the PSC within 4.5 h of stroke symptom onset. Here, *n* = 132/164 patients (80.5%) did not receive prior oral anticoagulation treatment, of whom *n* = 58 (43.9%) received IVT (see Figure 2). In comparison, *n* = 32/164 patients (19.5%) of potentially eligible patients were under oral anticoagulation treatment (VKA, *n* = 14; NOAC, *n* = 18). One patient under prior treatment with VKA received IVT at the local PSC and one patient under prior NOAC treatment received IVT after being transferred to the CSC, where NOAC-specific coagulation testing was performed. Common reasons for withholding treatment in the nonanticoagulation group were nondisabling symptoms (*n* = 27) and an extended time window without the possibility for advanced multimodal imaging examination, which were revealed after taking the full history during the teleneurological consultation (*n* = 14) (Figure 2). For *n* = 11/30 patients (37%) under prior treatment with oral anticoagulants who were otherwise found to be potentially eligible but did not receive IVT, NOAC treatment was the only documented reason for withholding treatment. NOAC-specific coagulation testing was available in three of these patients (all concentrations ≥ 30 ng/mL).

### 3.4. Adaptation of Oral Anticoagulation after Stroke

In *n* = 35/63 patients (56%), oral anticoagulation therapy was discontinued for less than 3 days (see Table 3). The prior therapeutic regimen was modified in more than half of the patients (*n* = 33), the most common changes being to prescribe NOAC instead of VKA (*n* = 12) and to adapt the NOAC dosages (*n* = 8).

## 4. Discussion

We investigated the treatment of AIS in orally anticoagulated patients in a teleneurological network. Two major findings emerged from our study: Firstly, AIS under oral anticoagulation treatment represents a frequent reason for consultation and, secondly, local treatment options are limited, with more than one-fifth of patients requiring interhospital transfer in an acute situation for further diagnostic testing and treatment evaluation.

While 17.5% of all patients with AIS were on oral anticoagulation treatment at the time of presentation, of whom 32 (51%) presented within 4.5 h after symptom onset, only 2 orally anticoagulated patients (6%) actually received thrombolytic treatment. Indeed, prior NOAC intake represented a major obstacle in administering IVT. These findings are in line with data from conventional in-person settings [3,5].

According to our observations, patients under prior oral anticoagulation therapy tended to be transferred more frequently to another hospital in an acute situation, while overall rates of final endovascular treatment were similar to those for patients who did not receive prior treatment with oral anticoagulants. These findings are surprising, as evaluation of potential endovascular treatment was among the underlying reasons in all orally anticoagulated patients who were transferred in an acute situation. However, vascular imaging had only been conducted in 40% of patients before being transferred, in some because CT angiography was not available at the local PSC and in others because immediate transfer without waiting to perform CT angiography was preferred. Lower thresholds for transferring orally anticoagulated patients due to limited local treatment options could be a potential reason and might result in higher rates of futile or noneligible transfers. Due to the observational nature of our study, we cannot prove that prior oral anticoagulation treatment actually caused more frequent secondary transportation during the acute phase of stroke. However, local treatment options were limited in patients who had previously received NOAC treatment: Neither specific quantitative coagulation testing nor reversal agents were available at teleneurological spoke centers, rendering interhospital transfer to the CSC the only option to evaluate acute reperfusion therapy. Meanwhile, in patients on dabigatran, we are gaining experience in using the antagonist idarucizumab so that IVT can be administered [4,12,13]. Indeed, in the last few years, some of the local centers have performed IVT after administering idarucizumab (launched in Germany in January 2016) to selected patients who had previously received dabigatran, guided by our teleneurological advice. In addition, since 2019, one of the spoke clinics implemented point-of-care testing, making it possible to initiate thrombolysis using Hemochrome Signature Elite^®^ in conjunction with thresholds authorized in the 2018 Karolinska Stroke Update [7].

Considering changes in demographics, the increased use of NOAC, and globally expanding teleneurological services, it is likely that AIS under oral anticoagulation therapy in the setting of teleneurology will also gain in importance in the future. Therefore, local diagnostic strategies allowing for patient selection at remote teleneurological spoke centers must be improved. Although laboratory-based NOAC drug monitoring can be performed and results received rapidly at specialized centers [14], this approach does not seem feasible for lower-volume, remote hospitals. Point-of-care testing might offer a potential solution, although this is currently not widely available for NOACs.

If rapid testing is not available locally, prehospital triage starting at the dispatch center should include asking about therapy with oral anticoagulants, which, if affirmative, would trigger direct transport to centers with available test capacities in order to reduce times to reperfusion treatment.

We conducted a retrospective analysis here, and some limitations should be considered. As all data were collected during routine clinical examinations, important parameters of stroke treatment such as modified Rankin scale score at three months after stroke were not assessed systematically. Despite screening 1500 teleneurological visits, the number of consultations with a final diagnosis of ischemic stroke was lower than expected, resulting in a limited sample size of patients who suffered AIS while on treatment with oral anticoagulants. Nevertheless, we found that approximately one-fifth of the patients with AIS were taking oral anticoagulants. Given that more than 35,000 teleneurological consultations are performed in Germany per year [15], this number represents an important cohort in telemedical consultations.

## 5. Conclusions

In conclusion, to our knowledge, this is the first study to focus on the management of AIS in orally anticoagulated patients in the setting of teleneurology. We provide real-world data from a large geographic area in southwestern Germany, showing that AIS under oral anticoagulation treatment is a common reason for teleneurological consultation. Future studies should focus on patient selection criteria and the development of specific therapeutic strategies that are feasible for teleneurological spoke centers, where local diagnostic and treatment options can be improved.

## Figures and Tables

**Table 1:** Characteristics of patients with ischemic stroke categorized according to anticoagulation status at admission (*n* = 359).

	No Prior OAC *n* = 296	Prior OAC *n* = 63	*p*-Value
Age	75.4 (64.1–81.6)	79.8 (75.3–85.3)	<0.001
Female sex	146 (49.3)	36 (57.1)	0.270
Medical history			
Atrial fibrillation	34 (11.5)	53 (84.1)	<0.001
DVT or PE	8 (2.7)	13 (20.6)	<0.001
Malignancy	24 (8.1)	15 (23.8)	0.001
Mechanical heart valve	1 (0.3)	1 (1.6)	0.321
Arterial hypertension	212 (71.6)	49 (77.8)	0.354
Diabetes mellitus	91 (30.7)	19 (30.2)	>0.99
Hyperlipidemia	84 (28.4)	16 (25.4)	0.757
Ischemic heart disease	51 (17.2)	18 (28.6)	0.052
Peripheral artery disease	30 (10.1)	10 (16.1)	0.185
Stroke/TIA	73 (24.7)	23 (36.5)	0.061
Prior medication			
Antiplatelet	117 (39.5)	7 (12.7)	<0.001
VKA	-	24 (38.0)	-
NOAC	-	39 (62.0)	-
Functional status			
Premorbid mRS ^a^	1 (0–3)	2 (1–3)	0.002
mRS at discharge ^b^	2 (1–4)	3 (2–4)	0.018
NIHSS at admission ^c^	4 (2–9)	6 (3–13)	0.014
NIHSS at discharge ^d^	1 (0–3)	2 (1–6)	0.053
Onset to admission at PSC, hours ^e^	4.2 (1.4–10.5)	2.4 (1.2–6.8)	0.067

Data are *n* (%) or median (IQR). Abbreviations: OAC, oral anticoagulation; DVT, deep vein thrombosis; PE, pulmonary embolism; TIA, transient ischemic attack; VKA, vitamin K antagonist; NOAC, non-VKA oral anticoagulant; mRS, modified Rankin scale; NIHSS, National Institutes of Health Stroke Scale; PSC, primary stroke center. Data available in ^a^ *n* = 293 (no prior OAC), *n* = 60 (prior OAC); ^b^ *n* = 265, (no prior OAC), *n* = 53 (prior OAC); ^c^ *n* = 293 (no prior OAC), *n* = 63 (prior OAC); ^d^ *n* = 237 (no prior OAC), *n* = 51 (prior OAC); ^e^ *n* = 133 (no prior OAC), *n* = 32 (prior OAC).

**Table 2:** Management of patients with ischemic stroke categorized according to anticoagulation status at admission (*n* = 359).

	No Prior OAC *n* = 296	Prior OAC *n* = 63	*p*-Value
Large-vessel occlusion	48 (16.2)	9 (14.3)	0.850
Imaging modality at admission			
CT	290 (98.0)	62 (98.4)	>0.99
MRI	6 (2.0)	1 (1.6)	>0.99
Acute vascular imaging	55 (18.6)	16 (25.4)	0.225
Acute Management			
Acute interhospital transfer	41 (13.9)	15 (23.8)	0.056
IVT	60 (20.3)	2 (3.2)	<0.001
EVT	22 (7.4)	8 (12.7)	0.207
Admitting ward			
General ward	9 (3.0)	1 (1.6)	>0.99
Stroke unit	249 (84.1)	56 (88.9)	0.438
Intensive care unit	36 (12.2)	6 (9.5)	0.669
Complications			
Any complication	51 (17.2)	11 (17.5)	>0.99
Pneumonia	21 (7.1)	7 (11.1)	0.300
Intracerebral hemorrhage	9 (3.0)	2 (3.2)	>0.99
Malignant infarction	13 (4.4)	-	0.136
Discharge to			
Patient’s home	108 (36.5)	20 (31.7)	0.563
Rehabilitation unit	112 (37.8)	23 (36.5)	0.887
Nursing home	21 (7.1)	8 (12.7)	0.199
Other hospital	33 (11.1)	12 (19.0)	0.095
Length of stay in days	6 (3–10)	6 (3–13)	0.201

Data are *n* (%) or median (IQR) if not indicated otherwise. Abbreviations: CT, computed tomography; MRI, magnet resonance imaging; IVT, intravenous thrombolysis; EVT, endovascular therapy; mRS, modified Rankin scale; NIHSS, National Institutes of Health Stroke Scale.

**Table 3:** Anticoagulation after stroke.

Treatment Interruption in Days *	
≤3	35 (66)
4–12	11 (21)
>12	7 (13)
Treatment modifications ^#^	
No treatment modifications	30 (48)
Adaptation of NOAC dosage	8 (13)
VKA to NOAC	12 (19)
NOAC to VKA	2 (3)
NOAC to different NOAC	4 (6)
Parenteral anticoagulation	3 (5)
No further anticoagulation	4 (6)

Data are *n* (%). Abbreviations: VKA, vitamin K antagonist; NOAC, non-VKA oral anticoagulant. Information was available for * 53 patients, and ^#^ 63 patients.

## Data Availability

Data are available upon reasonable request from the corresponding author.

## Appendix A

**Table A1:** Characteristics of patients with ischemic stroke presenting within 4.5 h after onset of symptoms categorized according to anticoagulation status at admission (*n* = 164).

	No Prior OAC *n* = 132	Prior OAC *n* = 32	*p*-Value
Age	74.4 (63.9–81.8)	77.6 (71.9–85.1)	0.041
Female sex	64 (48.5)	18 (56.3)	0.555
Medical history			
Atrial fibrillation	21 (15.9)	26 (81.3)	<0.001
DVT or PE	4 (3.0)	8 (25.0)	<0.001
Malignancy	13 (9.8)	10 (31.3)	0.004
Mechanical heart valve	1 (0.8)	1 (3.1)	0.353
Arterial hypertension	88 (66.7)	30 (93.8)	0.002
Diabetes mellitus	40 (30.3)	7 (21.9)	0.391
Hyperlipidemia	31 (23.5)	7 (21.9)	>0.99
Ischemic heart disease	18 (13.6)	9 (28.1)	0.062
Peripheral artery disease	17 (12.9)	6 (18.8)	0.400
Stroke/TIA	31 (23.5)	14 (43.8)	0.028
Prior medication			
Antiplatelet	50 (37.9)	3 (9.3)	0.007
VKA	-	14 (43.8)	-
NOAC	-	18 (56.2)	-
Functional status			
Premorbid Mrs ^a^	1 (0–3)	2 (1–3)	0.066
mRS at discharge ^b^	2 (1–4)	3 (2–4)	0.086
NIHSS at admission ^c^	5 (2–10)	6 (2–13)	0.350
NIHSS at discharge ^d^	1 (0–4)	3 (0–6)	0.263

Data are *n* (%) or median (IQR). Abbreviations: OAC, oral anticoagulation; DVT, deep vein thrombosis; PE, pulmonary embolism; TIA, transient ischemic attack; VKA, vitamin K antagonist; NOAC, non-VKA oral anticoagulant; mRS, modified Rankin scale; NIHSS, National Institutes of Health Stroke Scale. Data available in ^a^ *n* = 132 (no prior OAC), *n* = 31 (prior OAC); ^b^ *n* = 121 (no prior OAC), *n* = 26 (prior OAC); ^c^ *n* = 132, (no prior OAC), *n* = 32 (prior OAC); ^d^ *n* = 109 (no prior OAC), *n* = 25 (prior OAC).

**Table A2:** Management of patients with ischemic stroke presenting within 4.5 h after onset of symptoms categorized according to anticoagulation status at admission (*n* = 164).

	No Prior OAC *n* = 132	Prior OAC *n* = 32	*p*-Value
Large vessel occlusion	31 (23.5)	3 (9.4)	0.091
Acute vascular imaging	38 (28.8)	8 (25.0)	0.827
Acute Management			
Acute interhospital transfer	28 (21.2)	7 (21.9)	>0.99
IVT	58 (43.9)	2 (6.3)	<0.001
EVT	15 (11.4)	2 (6.3)	0.530
Admitting ward			
General ward	3 (2.3)	1 (3.1)	0.584
Stroke unit	108 (81.8)	28 (87.5)	0.603
Intensive care unit	20 (15.2)	3 (9.4)	0.572
Complications			
Any complication	28 (21.2)	7 (21.9)	>0.99
Pneumonia	13 (9.8)	5 (15.6)	0.351
Intracerebral hemorrhage	6 (4.5)	1 (3.1)	>0.99
Malignant infarction	5 (3.8)	-	0.584
Discharge to			
Patient’s home	46 (34.8)	9 (28.1)	0.536
Rehabilitation unit	47 (35.6)	12 (37.5)	0.840
Nursing home	11 (8.3)	6 (18.8)	0.104
Other hospital	15 (11.4)	5 (15.6)	0.548
Length of stay in days	5 (3–9)	9 (3–18)	0.011

Data are *n* (%) or median (IQR) if not indicated otherwise. Abbreviations: IVT, intravenous thrombolysis, EVT, endovascular therapy.

**Table A3:** Logistic regression model for performing acute interhospital transfer

Variables	OR	95% CI	*p*-Value
Age	0.994	0.96–1.029	0.732
Premorbid mRS	0.428	0.3–0.611	<0.001
NIHSS at admission	1.248	1.172–1.318	<0.001
History of malignancy	1.359	0.458–4.029	0.58
Prior OAC treatment	2.306	0.958–5.552	0.062

mRS, modified Rankin scale; NIHSS, National Institutes of Health Stroke Scale; OAC, oral anticoagulation; OR, odds ratio; CI, confidence interval.
